# Translanguaging in conversations for people with aphasia living in Greater Johannesburg, South Africa

**DOI:** 10.4102/sajcd.v72i1.1082

**Published:** 2025-02-12

**Authors:** Mellissa Bortz, Mira Goral

**Affiliations:** 1 Department of Communication Sciences and Disorders, St John’s University, Queens, United States; 2 Department of Speech- Language-Hearing Sciences, Lehman College, The City University of New York, Bronx, United States

**Keywords:** aphasia, translanguaging, multilingual, narratives, group conversation, assessment, South Africa

## Abstract

**Background:**

Challenges associated with language assessment in multilingual people with aphasia include the lack of linguistically and culturally appropriate assessment tools. Moreover, most multilingual people with aphasia are assessed in each of their languages separately. However, many multilingual people use elements from their complete linguistic repertoire rather than communicate in one language at a given conversation.

**Objectives:**

We aimed to examine language production in multilingual speakers with aphasia within a translanguaging approach to assessment, that is, without specifying a single target language. Our four research questions inquired about the characteristics of translanguaging in elicited language production and about the influence of task, topic and individual variables on translanguaging patterns.

**Method:**

We elicited individual monologues and group conversations from seven people with aphasia living in the Johannesburg Metropolitan Municipality in South Africa. We coded their language output in terms of the number of words used and the languages selected.

**Results:**

Participants used translanguaging to varying degrees. Five participants used both isiZulu and English in their responses; two participants each used only one language (isiZulu or English). Topic and context of conversation did not seem to affect the pattern of language use.

**Conclusion:**

Seven multilingual people with aphasia demonstrated the use of translanguaging during elicited language testing. An assessment procedure that allows for the use of multiple languages without restricting the conversation to one language is a feasible approach to assessing people from multilingual communities.

**Contribution:**

The study introduces an alternative approach to assessing multilingual people with aphasia and demonstrates its feasibility.

## Introduction

Stroke is a leading cause of disability worldwide (Odendaal & Tönsing, 2024; Prust et al., 2024). Stroke results in devastating effects on those who survive it, leading to communication, physical, social and psychological challenges (Cherney et al., 2024; Dickey et al., 2010). Communication challenges due to aphasia occur in 30% – 38% of clients (Shiggins et al., 2024). Aphasia negatively affects language comprehension and production (Cherney et al., 2024) and is often concomitant with other communication challenges such as alexia, agraphia, acalculia and dysarthria (Hinckley, 2016; Kaylor & Singh, 2023; Ramos-Lima et al., 2018). People with aphasia can improve their communication abilities, especially if assessment and rehabilitation are provided by speech-language therapists (Akinyemi & Adeniji, 2018; Brady et al., 2016).

Unlike in Minority World Countries, in which the rates of stroke have decreased recently by 42% (Prust et al., 2024; Roushdy et al., 2022), in Majority World Contexts^1^ the rates of stroke are growing more than 100%, and this number is predicted to increase in the future (Feigin et al., 2021; Mensah et al., 2015; Roushdy et al., 2022). Reasons for this increase include challenges in providing prevention, assessment and treatment of stroke. Currently, Africa has one of the highest numbers of strokes in the world even though less than a century ago stroke was unknown in Africa (Akinyemi et al., 2021). In South Africa, stroke is the third leading cause of death (Institute for Health Metrics and Evaluation, 2019). A systematic review of prevalence of stroke in South Africa revealed an increase of 25% from 2000 to 2016 (Abedlatif et al., 2021). Further, there is very little rehabilitation and speech-language therapy that take place after a stroke in Africa (Akinyemi et al., 2021). In much of Africa, patients receive short periods of rehabilitation such as physical and speech-language therapy, when available, but are then discharged to the care of their families. Bernhardt et al. (2020) found that doctors sometimes have limited knowledge, and patients do not receive rehabilitation or have to wait long periods from the onset of stroke until rehabilitation begins.

It is important to perform assessment in aphasia in order for speech-language therapy to begin as soon as possible, to maximise the spontaneous recovery period (Bernhardt et al., 2017; Mattioli et al., 2014). Multilingual people who have aphasia typically experience impairments in all their languages, although patterns of greater or lesser relative impairment in one of the languages have been reported (Goral & Lerman, 2020). Aphasia assessment of multilingual people is used to determine not only the extent and type of impaired and preserved abilities in each language, but also in which language to provide therapy, because this factor influences the effectiveness of speech-language therapy (Goral & Lerman, 2020; Goral et al., 2023; Grasemann et al., 2021). Currently, assessment is typically conducted in one language only or in several of a multilingual person’s languages, separately. A challenge is that the majority of South African speech-language therapists are monolingual, and even those therapists who are multilingual are unable to speak all approximately 35 languages and varieties in South Africa. South Africa has 12 official languages (isiZulu, isiXhosa, isiNdebele, siSwati, Sesotho sa Leboa^2^ SeSotho, Setswana, Xitsonga, Tshivenda, South African Sign language, English and Afrikaans) and numerous unofficial languages, dialects and pidgins, such as Tsotsitaal^3^ (Makalela, 2022; Molamu, 1995), Sepitori (Ditsele & Mann, 2014; Wagner et al., 2020) and Soweto Zulu (Bortz, 2023). isiZulu and English are the lingua francas of the country, with almost 25% of the population speaking isiZulu as a first language according to the 2022 South African Census. The Nguni group of languages consists of isiZulu, isiXhosa, Seswati and Ndbele and the Sotho group consists of Northern Sotho, Southern Sotho and Setswana; the languages within these groups are mutually intelligible.

A further challenge for the South African speech-language therapist is that there are a limited number of aphasia assessments available in South African languages. The lack of appropriate multilingual and/or culturally and linguistically diverse language assessment materials, which can lead to inaccurate diagnosis, can result in the abrogation of the Universal Declaration of Linguistic Rights Articles 3.1 and 3.2, which state that there is a right to the use of one’s own language both in private and in public, the right for their own language and culture to be taught. To illustrate, the one aphasia test that was designed for the purpose of assessing multilingual individuals, the Bilingual Aphasia Test (BAT) (Paradis, 1987), has versions in more than 70 languages but none in any of the South African languages. The adaptation and validation of the BAT to each of the nine South-eastern Zone Bantu languages (SeBZ), for example, would involve carefully generating language-specific versions of the test, and testing multilingual people with aphasia (PWA) in each language that they speak, and would therefore be extremely time-consuming.

To overcome the myriad challenges associated with translating and adapting structured aphasia tests (Goral & Galletta, 2024; Martínez-Ferreiro et al., 2024), clinicians can opt to conduct assessments of elicited connected language production and conversations (also referred to as connected speech production, or discourse, e.g., Stark et al., 2021), which are relatively easily adapted to any language and culture (Best et al., 2016; Kong, 2024; Stark et al., 2021). Assessments of discourses in aphasia have been successfully used in South Africa. Mosdell et al. (2010) used a version of Cookie Theft picture from the Boston Diagnostic Aphasia Examination (Goodglass et al., 2000) to test speakers of isiXhosa and Afrikaans; Penn and Beecham (1992) used elicited discourse to assess and to treat a multilingual client, a speaker of, among others, Ndebele, Pedi, isiZulu, English, Afrikaans, Xhosa and South Sotho.

It may be unrealistic to assess multilingual speakers in each of their languages from a monolingual perspective of a single target language, be it with structured tests or elicited production (Khamis & Bortz, 2023). An alternative to the series of single-language assessments of each of the languages of multilingual people is a translanguaging approach of language assessment (Fei & Weekly, 2022; García, 2009). Translanguaging is a multidisciplinary approach, drawing from fields such as linguistics, education, psychology and sociolinguistics (Bonancina-Pugh et al., 2021; Li & García, 2022). The approach fosters inclusive and effective communication across diverse linguistic landscapes, embracing the use of multiple languages within a single interaction. It captures the dynamic and flexible nature of language use in multilingual settings, and thus offers a powerful tool for discourse and conversation analysis (Fei & Weekly, 2022; García, 2009; Khamis & Bortz, 2023). A main contribution of translanguaging is the inclusion of the individual’s full, inclusive, integrated and unique language repertoire, instead of considering each language separately (Cenoz & Goerter, 2020; Otheguy et al., 2015; Sefotho, 2022; Simpson, 2017; Xin et al., 2021). It thus can offer a socially just, equitable assessment for multilingual clients (Cioè-Peña & Snell, 2015) and a tool to handle multilingual persons’ common practice of using more than one language when communicating (MacSwan, 2022; Myers-Scotton, 1997). The discussion of translanguaging has stirred some controversies in the literature (Treffers-Daller, 2024), as some have considered translanguaging to be superior to previous terms (such as code mixing, code switching), although it does not have to be taken as a replacement for such terms (Li, 2018).

A key tenet of translanguaging is that language does not conform to socio-political boundaries of named countries (García, 2009; García & Lin, 2017; García & Li, 2014; Otheguy et al., 2015), which may be particularly relevant to Africa. For example, a named language such as Ndebele is spoken in both South Africa and Zimbabwe. Translanguaging can also accommodate the flexible ways in which multilingual individuals draw on multiple languages to enhance communicative potential (Simpson, 2017) and the fluidity that exists between languages (Makalela, 2022; Sefotho, 2022), referred to as *Ubuntu* translanguaging. *Ubuntu* translanguaging refers to an African value system of ‘I am because you are and you are because I am’ (Makalela, 2022). Dauda (2017) describes *Ubuntu* and *Omolúwàbí*^4^ as long-standing indigenous systems of African humanism and ethical governance which are related to democracy, equity,moral responsibility, and are self-regulatory. *Ubuntu* translanguaging offers a non-Western decolonial pedagogical model where African languages and English are equally used in all classrooms for all subjects. In fact, translanguaging has been used in education very successfully in South Africa from primary school through to higher education (Makalela, 2022). South Africa is one of the five countries designated as a research centre for translanguaging (Xin et al., 2021).

Developments in bilingual assessment in the profession of speech-language therapy have focused on highlighting the need to evaluate the two languages to inform the selection of language for intervention (Goral & Lerman, 2020). These developments remain centred on separate use of the linguistic systems, with growing acknowledgement of dialects and genres beyond named nation-state languages. To date, the profession has not yet promoted exploring and designing assessments and interventions that embrace translanguaging in their design, coding, or analysis. The rationale for this study was therefore to explore the feasibility of a translanguaging-based approach to assessment in aphasia. To this end, Bortz and Goral (manuscript submitted for publication) have conducted a study in an effort to characterise the translanguaging patterns of South African adults from the City of Johannesburg Metropolitan Municipality and to determine how conversational context, topic and multilingualism profile (age of language acquisition, frequency of language use and self-rated proficiency) predicted translanguaged discourse observed in healthy young and older adults. Using elicited language production tasks and bilingual background questionnaires, the authors demonstrated that all the participants, who were speakers of a number of South African languages, made use of translanguaging, to varying degrees. The results demonstrated that translanguaging is a useful method for characterising the unique aspects of South African discourse, and may be applied to other multilingual and neurodiverse communities. Here, we aimed to determine the translanguaging profiles of people with aphasia in the City of Johannesburg Metropolitan Municipality in South Africa, using the same procedure used in Bortz and Goral (manuscript submitted for publication). If feasible, this approach may offer a novel, ecologically valid framework for assessing multilingual individuals with acquired communication impairments.

The following research questions guided our study:

1. *Is testing multilingual people with aphasia within a translanguaging approach feasible?*
2. *What are the characteristics of translanguaging in elicited language production of people with aphasia in the greater Johannesburg Metropolitan Municipality, South Africa?*
3. *Are there differences in the translanguaging behaviour between narratives and group conversations, and based on the topic of conversation?*
4. *Do characteristics of language acquisition and use, and of self-reported language mixing affect translanguaging?*

## Research methods and design

### Study design

This is a single-subject (case series) study, using mixed (quantitative and qualitative) methods, reporting descriptive data from individual cases (Orlikoff et al., 2014).

### Setting

Data collection took place in the City of Johannesburg Metropolitan Municipality, South Africa.

### Participants

Seven participants with aphasia took part in the study. The participants ranged in age from 25 years to 75 years (mean 41 years^5^). They were six women and one man. Table 1 provides information about the participants’ age, gender, education, and information about the aetiology and presentation of aphasia. The inclusion criteria for participation in this study were having sustained a stroke at least 6 months before testing began and being a speaker of two or more South African languages. This was a convenient sample, and participants were recruited through word-of-mouth via the research assistant clinician. All participants lived within the City of Johannesburg Metropolitan Municipality. Four of the participants (Participant 2, Participant 3, Participant 6 and Participant 7) were born in Soweto or Johannesburg (Gauteng), which are multilingual urban areas (Bortz, 2023). Participant 1 and Participant 5 were originally from KwaZulu-Natal, and Participant 4 was from the Eastern Cape (see Table 1). Participants’ language background information is presented in Table 2.

**TABLE 1:** Participant information.

Participant	1	2	3	4	5	6	7
Age	27	75	40	37	41	40–50	25
Gender	Female	Female	Female	Female	Female	Male	Female
Aetiology	Left CVA	Left CVA	Left CVA	Left CVA	Left CVA	Left CVA	Left CVA
Years post onset	12	1	1	2	1	2	5
Presentation	Expressive aphasia; moderately severe	Expressive language impairment and cognitive impairment	Expressive language impairment	Expressive language aphasia, no physical impairment, motor speech disorder (dysarthria)	Moderate mixed aphasia	Moderate aphasia with right lower limb impairment	Moderate mixed aphasia
Education (in years)	17	20	12	11	13	16	11
Hometown	Umlazi, KwaZulu-Natal	Johannesburg	Dobsonville, Soweto	Eastern Cape	KwaZulu-Natal	Diepkloof, Soweto	Soweto
Current town	Soweto, Johannesburg	Johannesburg	Dobsonville, Soweto	Chiawelo, Soweto	Orlando, Soweto	Witpoortjee, Roodepoort	Soweto
Home language	isiZulu	English	isiZulu	isiXhosa	isiZulu	Setswana	isiZulu
Current language(s)	Predominately uses English to express herself but can understand isiZulu	English	isiZulu and English	isiZulu and English	isiZulu and English	English	English
Problem statement	She had stroke when in grade 10, secondary school. She had to drop out and was unable to complete her schooling. She is physically impaired with non-functioning right limbs. She prefers communicating in English because she easily remembers or accesses her English lexicon as compared to isiZulu and other languages (Sepedi only comprehend, isiXhosa).	Stroke has impacted her social life, as she prefers not to speak often and avoids social situations. She attended an English and Afrikaans school but currently uses and remembers English better than Afrikaans because she only used Afrikaans at school. Other language exposed to is Hebrew but only understand it.	Knows and uses various languages (isiXhosa, Sotho, Setswana, English, Afrikaans). She also attended an English and Afrikaans school. As a result, she prefers to communicate in English than any other languages.	isiXhosa speaker but uses isiZulu because people around her use isiZulu	She indicated challenges performing her work duties optimally as well as driving. She indicated knowledge of English, Afrikaans, isiZulu, isiXhosa, Setswana (only comprehend). Proficiency of isiZulu language was noted with English use. Left hemisphere lesion with no physical impairments.	Languages exposed to are Setswana, Sesotho, isiZulu, English and Afrikaans, but currently prefers to use English as it seems easier than his language.	Languages exposed to in the past: Isizulu Setswana, Sesotho, English. Could only use English because isiZulu was difficult to recall during interactions.

CVA, cerebrovascular accident.

**TABLE 2:** Participants’ language background.

Participant	1	2	3	4	5	6	7
1st acquired language	IsiZulu	English	isiZulu	isiXhosa	IsiZulu	Setswana	isiZulu
Age of acquisition	0	0	< 5	0	0	0	2
Pre-stroke proficiency/post stroke s.r. proficiency, comprehending (max 10)	10/8	10/8	10/7	10/10	10/7	10/10	10/NA
Pre-stroke proficiency/post stroke s.r. proficiency, speaking (max 10)	8/8	10/3	10/7	10/8	10/8	7/10	10/NA
Pre-stroke proficiency/post stroke s.r. proficiency, reading (max 10)	8/7	10/5	10/8	10/5	10/4	7/10	10/NA
Pre-stroke proficiency/post stroke s.r. proficiency, writing (max 10)	8/6	10/3	10/7	10/10	10/0	10/9	10/NA
Later acquired language	English	Afrikaans	English	English	English	English	English
Age of acquisition	6	7	17	9	6	6	6
Pre-stroke proficiency/post stroke proficiency, comprehending (max 10)	10/9	10/2	10/6	10/8	10/7	10/3	NA
Pre-stroke proficiency/post stroke s.r. proficiency, speaking (max 10)	8/7	10/0	10/7	10/7	10/6	10/3	NA
Pre-stroke proficiency/post stroke s.r. proficiency, reading (max 10)	10/6	10/0	4/3	10/9	10/8	10/9	NA
Pre-stroke proficiency/post stroke s.r. proficiency, writing (max 10)	9/8	10/0	1/3	10/10	10/6	10/9	NA
%Language use	70% English 20% isiZulu 10% isiXhosa	70% English 20% Afrikaans 10% Hebrew	NA	30% isiZulu 20% isiXhosa 10% English	40% English 20% isiZulu 20% Setswana 10% isiXhosa 10% Afrikaans	80% Setswana 20% English	100% English
Frequency of mixing (max 60)	44	15	50	42	54	49	49

s.r., self-reported; NA, not applicable.

### Ethical considerations

The study was approved by the Institutional Review Board (IRB) of the City University of New York (2023-0798-Lehman), which served as the IRB of record. All participants signed a consent form after being recruited. The study procedure was discussed with the participants, and they indicated their understanding and provided their consent. The IRB process helps ensure participants’ rights for respect and confidentiality. Participants were reimbursed for their time using an airtime gift card.

## Data collection

### Materials

#### Language questionnaires

Two language background questionnaires were used: the Language Experience and Proficiency Questionnaire (LEAP-Q) (Marian et al., 2007) and the Bilingual Switching Questionnaire (BSWQ) (Rodriguez Fornells et al., 2012). The questionnaires were translated and adapted from English into Xitsonga, Sesotho, and isiZulu and administered to each participant in their first language. The LEAP-Q provides information about age, education, self-rated language proficiency and self-reported language use patterns in the languages that the participants spoke. The BSWQ elicits information about translanguaging habits (by asking participants about their habits of language mixing). Table 1 lists the languages each participant reported using; Table 2 indicates the first two languages that each participant spoke, their ages of acquisition, percentages of reported exposure and use and reported proficiency.

#### Narrative production

We used a modified version of the AphasiaBank protocol to elicit connected language production (MacWhinney et al., 2011). We modified it to accommodate the cultural and linguistic context of South Africans. We elicited personal narratives using the following topics: describing picture scenes of differently abled people socialising or of Nelson Mandela’s release from prison; how to make pap (a traditional South African dish); lobola (a bride price)/traditional/white wedding and coronavirus disease 2019 (COVID-19) recovery/stroke recovery journey. We elicited group conversations using the topics: Christmas, New Year, baking, news, sports and politics.

### Procedure

The examiner, a multilingual speech-language-therapist who speaks Xitsonga, Nguni languages (isiZulu and isiXhosa), Sotho languages (Sesotho, Setswana, Sepedi) and English, met with the individual participants and engaged in eliciting individual discourse. Free discourse was encouraged, and prompts were provided if needed. Individual narratives were elicited in an in-person meeting or via a video call between the examiner and each participant. Group discourse took place between the examiner and two participants. The examiner introduced the topic using translanguaging (i.e., multiple languages), and participants provided their opinions on the topic and a discussion among the group members ensued. Each participant produced narratives on three topics, and each group held conversations on three topics. If a participant did not contribute to the discourse, the examiner asked them if they had anything to add. Discourse was unstructured and participants spoke for as long as they wanted. Individual interactions ranged from 15 min to 30 min; group conversations ranged between 30 min and 75 min. The elicited language was audio-recorded for later transcription. In addition, each person completed the two questionnaires on language background (LEAP-Q and BSWQ) with the aid of the examiner.

### Transcription, coding and analysis of data

All recorded data were transcribed manually, and the total number of utterances and the total number of words per person per sample were counted. Whereas the elicitation approach used was within the translanguaging framework (Fei & Weekly, 2022), for the purpose of characterising language use among the participants, we counted the number of words produced in each language by each participant and reported the frequency distribution of words in each language per participant. Words that seem to be a blend of more than one language were coded as mixed. We note that there is great variability in the transliteration of the SeBZ languages, for example, isiZulu, and at times determining the language for each word was challenging. Due to the small, single-subject sample, we report descriptive statistics; we present both quantitative and qualitative results (Orlikoff et al., 2014).

## Results

Our first research question queried the feasibility of administering aphasia testing within a translanguaging framework. Qualitative evaluation suggests that it is possible to engage people with aphasia in elicited conversations by modelling and encouraging natural multiple-language use.

To answer our second research question, regarding the characteristics of translanguaging in elicited language production, we examined the relative frequency of language use in the elicited samples. The participants’ conversations ranged in length from 11 words to 328 words per person per sample, with a mean of 89 words for the individual production, and from 19 words to 104 words, with a mean of 43 for the group conversations (see Table 3). The participants used isiZulu and English to varying degrees in their connected speech production, with no other languages noted (see Table 4).

**TABLE 3:** Overall results.

Length	Individual monologues	Group conversations
Total words	1871	575
Mean	89.09	47.92
s.d.	72.08	26.81
Range	11–328	19–104

s.d., standard deviation.

**TABLE 4:** Translanguaging frequencies.

Participant setting	Topic	1		2		3			4			5			6		7	
		Z	E	Z	E	Z	E	E and Z	Z	E	E and Z	Z	E	E and Z	Z	E	Z	E
Individual	Picture description	100	0	0	100	44	56	-	0	100	-	77	23	-	21	79	13	87
	Stroke	NA	-	NA	-	68	32	-	50	50	-	92	8	-	61	39	61	39
	Making pap	NA	-	NA	-	75	25	-	69	31	-	86	14	-	82	18	NA	-
	Lobola	NA	-	NA	-	69	31	-	74	26	-	86	14	-	84	16	27	73
Group	Christmas	NA	-	NA	-	80	20	-	84	16	-	92	8	-	61	39	NA	-
	Politics or News	NA	-	NA	-	80	20	-	76	24	-	94	-	6	54	46	NA	-
	Baking	NA	-	NA	-	75	-	25	85	-	15	NA	-	-	NA	-	NA	-
	New Year	NA	-	NA	-	NA	-	-	NA	-	-	79	10.5	10.5	59	41	NA	-

E, English; Z, isiZulu; NA, not applicable.

To answer our third research question, regarding potential differences related to the setting and topic of conversation, we examined the participants’ languages in the individual and in the group conversations. In the picture description, one participant used isiZulu exclusively and two participants used English exclusively; the four participants who used both languages used either more isiZulu (Participant 5, 77%, Participant 6, 79%, Participant 7, 87%) or slightly more English (Participant 3, 56%). In the other three of the individual monologue topics, greater variation in language use was noted with frequencies ranging from 8% English (92% isiZulu, Participant 5 when talking about her stroke) to 50% English (Participant 4 when talking about her stroke). In the group conversations, the participants used isiZulu most, followed by English and a few instances of words blending English and isiZulu, with frequency of non-isiZulu words ranging from 6% (Participant 5 when discussing politics) to 46% (Participant 6 when discussing politics). Overall, it appears that the topic of conversation did not systematically affect the frequency of language use.

Finally, to address our fourth research question regarding individual characteristics, we examined the participants’ language background. The participants varied in their histories of language acquisition and use. Four participants (Participant 1, Participant 3, Participant 5, Participant 7) reported isiZulu to be their first acquired, home language. The remaining three each reported a different home language (isiXhosa, Setswana and English). Similarly, the participants varied in the languages they reported to have been using in the past and following the stroke (see Table 1 and Table 2). Five of the seven participants reported using additional languages, but all seven used isiZulu and English exclusively in the elicited conversations (Table 4).

The participants reported varying degrees of language mixing on the BSWQ. There was no clear relation between the reported mixing and the observed mixing, although Participant 2 who reported the least frequency of mixing on the questionnaire used only English in the one task she participated in. In contrast, Participant 1 used isiZulu only, but reported mixing her languages. No clear relationship emerged between reported language use prior to the stroke and the patterns observed in the participants’ outputs.

Qualitative examination of the translanguaging patterns revealed the use of English and isiZulu words as well as the adaptation of words from various origins into isiZulu. For instance, the production of the word ‘*bhaka*’ could originate from the English word ‘bake’, the Old English word ‘bacan’, or the Afrikaans word ‘*bak*’. Similarly, ‘Christmas’ was produced also as ‘*ikhisimusi*’. The English word ‘vote’ was adapted to ‘*ivoti*’, maintaining its original meaning while fitting into the linguistic structure of isiZulu. For example, Participant 6 mixed the word ‘Christmas’ and the isiZulu prefix *i* in: *ixmas*; the suffix *i* was added to the word ‘present’ in: ‘*presenti*’. Similarly, the English word ‘bake’ was inflected by Participant 3 as ‘*ukubake*’ and by Participant 4 as ‘*kubhakwama*’. Another example was noted by Participant 3 who produced the sentence: *ngibheka efonini amaupdates* [I look at the phone for updates]. Also, the participants used South African words such as ‘loadshedding’. Participants inserted single words and suffixes as well as whole phrases and sentences. For example, Participant 6 said when discussing politics:

*‘mhaunga bheka* most i i i party *legodi ledayo njee* there’s lot of things that is happening within for example *amacorruptioni nokuthi badli imali Zama* service delivery *ayisekho* sooo aaah according to what I understand or what I am looking forward to do now *ikuthi ngibalisile ukuyo voter ande ivote yami hisecrete*. I would advise each and everyone *kuthi ayovoter. Khonekuthi siendze* the right thing *masiyo bona kuthi ipart isileadayo yona* there are not doing their best, *kumele sovotele i parti e eyizo lethu uchico*.’ *[When you observe most of of of parties are stealing here there’s a lot of things that is happening within for example corruptions and spending money of service delivery is no longer available so aaah according to what I understand or what I am looking forward to do now is to register to vote and my vote is a secret. I would advise each and everyone to go vote. So that we do what’s right thing when we see the party that is leading us is not doing their best, we have to vote for a party that will bring change.] (Participant 6, 40–50, Male, Diepkloof, Soweto)*

In South Africa, the word ‘*yah*’ (or ‘*ja*’) is used irrespective of the language of conversation. Originally meaning ‘yes’ in Afrikaans, ‘*yah*’ is now commonly used in isiZulu, isiXhosa, English, and more. This fluid adoption highlights the dynamic and interconnected nature of South African communication, where linguistic boundaries are often blurred. Whether in urban or rural settings, ‘*yah*’ serves as a universal affirmation, reflecting the rich tapestry of cultural and linguistic influences in South Africa. Whereas some participants used the isiZulu word *yebo* [yes], all participants including the examiner used the word ‘*yah*’ multiple times in their conversations, as in *yah singa xubheka* [Yes, we can continue], produced by Participant 5.

## Discussion

In this study, we examined a novel approach to assessing language output from multilingual people with aphasia. Connected language production was elicited from seven multilingual people with aphasia living in City of Johannesburg Metropolitan Municipality, South Africa. We did not restrict the language of conversation; rather, encouraged participants to use their complete linguistic repertoire (Li & García, 2022). The participants did so to varying degrees. This manner of eliciting language production during assessment in aphasia is in contrast to the typical practice where each language of a multilingual person with aphasia is assessed separately (Goral et al., 2024). Our results demonstrated that this approach to testing is feasible and that people with aphasia do opt to use more than one language in their responses, thus providing an affirmative answer to our first research question. In response to our second research question, we observed that even though the participants in this study used only a portion of their complete linguistic repertoire, they typically did use more than one language. Of interest, the two languages the participants in this study selected to use in their conversations were the two languages used as lingua franca in their region (English and isiZulu). Most of the participants reported restricting their language production to one or two languages following the stroke. This is consistent with other multilingual people who reported reducing their linguistic repertoire and ‘sticking’ to one of their languages. Two of the participants, who were able to take part only in the picture description portion of the procedure, produced their responses in English only or isiZulu only. One participant reported that following the stroke, it was difficult for her to use more than one of her languages.

In response to our third research question, we did not find clear differences between translanguaging use patterns in the individual versus the group conversations, nor according to the topic of conversation. Although the length of the response varied by context, with longer responses evident in the individual compared to the group conversations, the proportions of language use did not. We did note, however, that the only instances where translanguaging was not used at all occurred during the picture description, with three participants using English or isiZulu exclusively. All other topics contained some degree of translanguaging.

In response to our fourth research question, concerning relevant variables affecting translanguaging behaviour, we found that regardless of the reported language history of the participants, all seven selected to use their isiZulu and/or English, and not their complete linguistic repertoire. These results point to a tenuous relationship between language history and translanguaging use, consistent with great variability typically found among multilingual people and their use of translanguaging (Fei & Weekly, 2020). Nor could we discern a clear pattern between aphasia severity and language use (Goral et al., 2019). All of the participants were classified as having moderate aphasia severity, yet they varied in the degree to which they used the two languages within a conversation.

In comparison to the group of neurologically healthy participants reported in Bortz and Goral (manuscript submitted for publication), the people with aphasia who participated in this study used translanguaging to a lesser degree. Whereas the neurotypical speakers used between two and four languages, the people with aphasia used two. It is possible that due to their aphasia and difficulty with language production, they restricted their language selection to two languages. Despite the quantitative differences, qualitatively the participants with and without aphasia demonstrated similar patterns of translanguaging. Both groups used morphemes, words, and sentences from more than one language within their utterances, and they used patterns of translanguaging that indicated ‘South Africanisms’ (such as *loadshedding* ‘rolling blackouts’ and *efonini amaupdates* [phone updates]). Although our sample of people with aphasia was small, translanguaging use was remarkably consistent among them, in that in comparison to neurologically healthy people, the people with aphasia used two of their multiple languages throughout the communication interactions in the study. Nevertheless, all participants used more than one language and may have benefitted from being able to do so.

### Limitations

A main limitation of this study is the small sample size and the limited data produced by the participants. The generalisability of the results beyond people with aphasia in the greater Johannesburg metropolitan area is limited, given the great variability in language use in South Africa. Furthermore, short language samples are typical of people with aphasia, especially with non-fluent aphasia (Faroqi-Shah, 2023), and additional data would be needed to corroborate the current findings. Another limitation is that some participants did not complete all tasks and could not reliably report about their complete language history. Finally, only one multilingual person transcribed the language samples. Any conclusions from our result should be drawn with caution. Nevertheless, the study demonstrated the importance of eliciting connected language production in a manner that allows multilingual people with aphasia to utilise their complete linguistic repertoire. Future efforts could be directed towards further analyses of the language produced in a translanguaging elicitation manner from multilingual people, including lexical choice, grammatical structure, and overall coherence of the narratives (Ed. Kong, 2024; Stark et al., 2021). Furthermore, to fully apply a translanguaging approach to the data, future linguistic analysis of the language samples within a translanguaging framework, that is, the evaluation of the accuracy and coherence of language output produced, regardless of the languages used (Goral et al., 2024), is warranted.

### Clinical implications

Speech-language therapists need to empower themselves to assess and treat multilingual and/or culturally and linguistically diverse clients with aphasia, for example, by familiarising themselves with different language families and language structures (Bortz, 2024) such as SeBZ languages and Indo-European languages. The use of elicited language production is one solution to the shortage of formal tests in the multiple languages spoken in South Africa. Using connected language and discourse in aphasia has been recognised as an important avenue to examine functional communication (e.g., Ed. Kong, 2024; Pritchard et al., 2018; Stark et al., 2021). Using a translanguaging framework for assessing clinical populations has the potential of mitigating some of the challenges associated with assessing multilingual people. This study also has implications for using translanguaging to conduct discourse-based therapy in aphasia, an approach that maximises person-centred intervention (Leaman & Archer, 2023).

## Conclusion

The results of this study show that a translanguaging approach to assessment has the potential as a valid language appraisal method for people with aphasia. All participants used more than one language in their communicative interactions, although they opted not to use their complete linguistic repertoire. Translanguaging offers a way in which multilingual and/or culturally and linguistically diverse clients can be assessed comprehensively and ultimately receive the urgent treatment that they need to maximise the potential for improvement in language challenges.

## Footnotes

1 Majority World Contexts are where the majority of the world’s population lives and where there may be resources, but these are controlled by Minority World Contexts, have their own resources but tend to have a monopoly on the world’s resources (Hyter & Salas-Provance, 2023). These terms are used for low- and middle-income countries and high-income countries, respectively.

2 These are the names used by the South African Census of 2022. Sepedi or Northern Sotho can also be used for Sesotho sa Lebeo.

3 Tsotsitaal is a language consisting of Afrikaans with other languages spoken in South Africa such as isiZulu. Molamu (1995) defines Tsotsitaal as a pidgin that developed among culturally and linguistically diverse speakers.

4 Same concept as *Ubuntu* used in Nigeria.

5 One participant declined to disclose his age.

## References

[CIT0001] Abdelatif, N., Peer, N., & Manda, S.O. (2021). National prevalence of coronary heart disease and stroke in South Africa from 1990–2017: A systematic review and meta-analysis. *Cardiovascular Journal of Africa*, 32(3), 156–160. 10.5830/CVJA-2020-045 33769427 PMC8756070

[CIT0002] Akinyemi, R.O., & Adeniji, O.A. (2018). Stroke care services in Africa: A systematic review. *Journal of Stroke Medicine*, 1(1), 55–64. 10.1177/2516608518775233

[CIT0003] Akinyemi, R.O., Ovbiagele, B., Adeniji, O.A., Sarfo, F.S., Abd-Allah, F., Adoukonou, T., Ogah, O.S., Naidoo, P., Damasceno, A., Walker, R.W., Ogunniyi, A., Kalaria, R.N., & Owolabi, M.O. (2021). Stroke in Africa: Profile, progress, prospects and priorities. *Nature Reviews Neurology*, 17(10), 634–656. 10.1038/s41582-021-00542-4 34526674 PMC8441961

[CIT0004] Bernhardt, J., Godecke, E., Johnson, L., & Langhorne, P. (2017). Early rehabilitation after stroke. *Current Opinion in Neurology*, 30(1), 48–54. 10.1097/WCO.0000000000000404 27845945

[CIT0005] Bernhardt, J., Urimubenshi, G., Gandhi, D.B.C., & Eng, J.J. (2020). Stroke rehabilitation in low-income and middle-income countries: A call to action. *Lancet*, 396(10260), 1452–1462. 10.1016/S0140-6736(20)31313-1 33129396

[CIT0006] Best, W., Maxim, J., Heilemann, C., Beckley, F., Johnson, F., Edwards, S.I., Howard, D., & Beeke, S. (2016). Conversation therapy with people with aphasia and conversation partners using video feedback: A group and case series investigation of changes in interaction. *Frontiers in Human Neuroscience*, 10, 562. 10.3389/fnhum.2016.00562 27872588 PMC5097900

[CIT0007] Bonacina-Pugh, F., Da Costa Cabral, I., & Huang, J. (2021). Translanguaging in education. *Language Teaching: Surveys and Studies*, 54(4), 439–471. 10.1017/S0261444821000173

[CIT0008] Bortz, M.A. (2023). Methods for devising a standardized language assessment for isiZulu pre-schoolers: Implications for Africa. In U.M. Ludtke, E. Kija, & M.K. Karia (Eds.), *Handbook of communication disabilities and language development in Sub-Saharan Africa* (pp. 423–440). Springer.

[CIT0009] Bortz, M.A. (2024). Introduction. In M.A. Bortz (Ed.), *A guide to global language assessment: A lifespan approach* (pp. 3–24). Routledge, Unpublished manuscript.

[CIT0010] Bortz, M.A., & Goral, M. (manuscript submitted for publication). Translanguaging in narratives and group conversations of South African adults. *International Journal of Speech Language Pathology*. 10.1080/17549507.2025.2544751 40833143

[CIT0011] Brady, M.C., Kelly, H., Godwin, J., Enderby, P., & Campbell, P. (2016). Speech and language therapy for aphasia following stroke. *Cochrane Database of Systematic Reviews*, 2016(6), CD000425. 10.1002/14651858.CD000425.pub4 27245310 PMC8078645

[CIT0012] Cenoz, J., & Gorter, D. (2020). Teaching English through pedagogical translanguaging. *World Englishes*, 39(2), 300–311. 10.1111/weng.12462

[CIT0013] Cherney, L.R., Kozlowski, A.J., Domenighetti, A.A., Baliki, M.N., Kwasny, M.J., & Heinemann, A.W. (2024). Defining trajectories of linguistic, cognitive-communicative, and quality of life outcomes in aphasia: Longitudinal observational study protocol. *Archives of Rehabilitation Research and Clinical Translation*, 6(2), 100339. 10.1016/j.arrct.2024.100339 39006119 PMC11240047

[CIT0014] Cioè-Peña, M., & Snell, T. (2015). Translanguaging for social justice. *Theory, Research, and Action in Urban Education*, 4(1), 1–5.

[CIT0015] Dauda, B. (2017). African humanism and ethics: The cases of Ubuntu and Omolúwàbí. In A. Afolayan & T. Falola (Eds.), *The Palgrave handbook of African philosophy* (pp. 475–491). Palgrave Macmillan.

[CIT0016] Dickey, L., Kagan, A., Lindsay, M.P., Fang, J., Rowland, A., & Black, S. (2010). Incidence and profile of inpatient stroke-induced aphasia in Ontario, Canada. *Archives of Physical Medicine and Rehabilitation*, 91(2), 196–202. 10.1016/j.apmr.2009.09.020 20159121

[CIT0017] Ditsele, T., & Mann, C.C. (2014). Language contact in African urban settings: The case of Sepitori in Tshwane. *South African Journal of African Languages*, 34(2), 159–165. 10.1080/02572117.2014.997052

[CIT0018] Faroqi-Shah, Y. (2023). A reconceptualization of sentence production in post-stroke agrammatic aphasia: The synergistic processing Bottleneck model. *Frontiers in Language Sciences*, 2, 1118739. 10.3389/flang.2023.1118739 39175803 PMC11340809

[CIT0019] Fei, Y., & Weekly, R. (2020). Examining the parameters of translanguaging in the context of Chinese bilinguals’ discourse practices. *International Journal of Multilingualism*, 19(3), 325–345. 10.1080/14790718.2020.1742721

[CIT0020] Feigin, V.L., Stark, B.A., Johnson, C.O., Roth, G.A., Bisignano, C., Abady, G.G., Abbasifard, M., Abbasi-Kangevari, M., Abd-Allah, F., Abedi, V., Abualhasan, A., Abu-Rmeileh, N.M., Abushouk, A.I., Adebayo, O.M., Agarwal, G., Agasthi, P., Ahinkorah, B.O., Ahmad, S., Ahmadi, S., Salih, Y.A. et al. (2021). Global, regional, and national burden of stroke and its risk factors, 1990–2019: A systematic analysis for the global burden of disease study 2019. *The Lancet Neurology*, 20(10), 795–820. 34487721 10.1016/S1474-4422(21)00252-0 PMC8443449

[CIT0021] García, O. (2009). *Bilingual education in the 21st century. A global perspective*. Wiley.

[CIT0022] García, O., & Lin, A.M. (2017). Translanguaging in bilingual education. In O. García, A.M.Y. Lin, & S. May (Eds.), *Bilingual and multilingual education* (3rd ed., pp. 117–130). Springer.

[CIT0023] Goodglass, H., Kaplan, E., & Barresi, B. (2000). *The Boston diagnostic aphasia examination*. Lippincott.

[CIT0024] Goral, M., & Galletta, E. (2024). Assessment in aphasia: Global perspectives. In M. Bortz (Ed.), *A guide to global language assessment: A lifespan approach* (pp. 307–321). Springer.

[CIT0025] Goral, M., & Lerman, A. (2020). Variables and mechanisms affecting response to language treatment in multilingual people with aphasia. *Behavioral Sciences*, 10(9), 144. 10.3390/bs10090144 32971777 PMC7551033

[CIT0026] Goral, M., Antolovic, K., Hejazi, Z., & Schulz, F. (2024). Using a translanguaging framework to examine language production in a trilingual person with aphasia. *Clinical Linguistics and Phonetics*, 39(1), 1–20. 38506332 10.1080/02699206.2024.2328240

[CIT0027] Goral, M., Norvik, M., & Jensen, B.U. (2019). Variation in language mixing in multilingual aphasia. *Clinical Linguistics & Phonetics*, 33(10–11), 915–929. 10.1080/02699206.2019.1584646 30836773 PMC6760868

[CIT0028] Goral, M., Norvik, M., Antfolk, J., & Lehtonen, M. (2023). Cross-language generalization in language treatment of multilingual aphasia: A meta-analysis. *Brain and Language*, 246, 105326. 10.1016/j.bandl.2023.105326 37994828

[CIT0029] Grasemann, U., Peñaloza, C., Dekhtyar, M., Miikkulainen, R., & Kiran, S. (2021). Predicting language treatment response in bilingual aphasia using neural network- based patient models. *Scientific Reports*, 11(1), 1. 10.1038/s41598-021-89443-6 34006902 PMC8131385

[CIT0030] Grönberg, A., Henriksson, I., & Lindgren, A.G. (2024). Long-term prognosis and health-related quality of life for people with Aphasia in Sweden. *Aphasiology*, 1–13. 10.1080/02687038.2024.232767838425351

[CIT0031] Hinckley, J.J. (2016). Aphasia practice in the year 2026. *Seminars in Speech and Language*, 37(3), 166–172. 10.1055/s-0036-1583546 27232092

[CIT0032] Hyter, Y.D., & Salas-Provance, M.B. (2023). *Culturally responsive practices in speech, language and hearing sciences*. Plural Publishing.

[CIT0033] International Journal of Speech-Language Pathology, M. (Forthcoming). *Translanguaging in narratives and group conversations of South African adults*. Unpublished manuscript. 10.1080/17549507.2025.2544751 40833143

[CIT0034] Kaylor, S.A., & Singh, S.A. (2023). Clinical outcomes associated with speech, language and swallowing difficulties post-stroke. *The South African Journal of Communication Disorders = Die Suid-Afrikaanse tydskrif vir Kommunikasieafwykings*, 70(1), e1–e15. 10.4102/sajcd.v70i1.957 PMC10623651 37916686

[CIT0035] Khamis, R., & Bortz, M.A. (2023). Translanguaging: A theoretical shift necessary for culturally and linguistically responsive practice in speech language pathology. In M. Goral & A. Lerman (Eds.), *Advances in the neurolinguistic study of multilingual adults: In honor of professor Loraine K. Obler* (pp. 36–50). Routledge.

[CIT0036] Kong, A. (Ed.). (2024). *Spoken discourse impairments in the neurogenic populations: A state-of-the-art, contemporary approach*. Springer Nature.

[CIT0037] Leaman, M., & Archer, B. (2023). Choosing discourse types that align with person-centered goals in aphasia rehabilitation: A clinical tutorial. *Perspectives of the ASHA Special Interest Groups*, 8(2), 254–273. 10.1044/2023_PERSP-22-00160

[CIT0038] Li, W. (2018). Translanguaging as a practical theory of language. *Applied Linguistics*, 39(1), 9–30. 10.1093/applin/amx039

[CIT0039] Li, W., & García, O. (2022). Not a first language but one repertoire: Translanguaging as a decolonizing project. *RELC Journal*, 53(2), 313–324. 10.1177/00336882221092841

[CIT0040] MacSwan, J. (Ed.). (2022). *Multilingual perspectives on translanguaging*. Multilingual Matters.

[CIT0041] MacWhinney, B., Fromm, D., Forbes, M., & Holland, A. (2011). AphasiaBank: Methods for studying discourse. *Aphasiology*, 25(11), 1286–1307. 10.1080/02687038.2011.589893 22923879 PMC3424615

[CIT0042] Makalela, L. (2022). *Not eleven languages: Translanguaging and South African multilingualism in concert*. De Gruyter Mouton.

[CIT0043] Marian, V., Blumenfeld, H.K., & Kaushanskaya, M. (2007). The Language Experience and Proficiency Questionnaire (LEAP-Q): Assessing language profiles in bilinguals and multilinguals. *Journal of Speech, Language and Hearing Research*, 50(4), 940–967. 10.1044/1092-4388(2007/067) 17675598

[CIT0044] Martínez-Ferreiro, S., Arslan, S., Fyndanis, V., Howard, D., Kraljević, J.K., Škorić, A.M., Munarriz-Ibarrola, A., Norvik, M., Peñaloza, C., Pourquié, M., Simonsen, H.G., Swinburn, K., Varlokosta, S., & Soroli, E. (2024). Guidelines and recommendations for cross-linguistic aphasia assessment: A review of 10 years of comprehensive aphasia test adaptations. *Aphasiology*, 1–25. 10.1080/02687038.2024.234345638425351

[CIT0045] Mattioli, F., Ambrosi, C., Mascaro, L., Scarpazza, C., Pasquali, P., Frugoni, M., Magoni, M., Biagi, L., & Gasparotti, R. (2014). Early aphasia rehabilitation is associated with functional reactivation of the left inferior frontal gyrus: A pilot study. *Stroke*, 45(2), 545–552. 10.1161/STROKEAHA.113.003192 24309584

[CIT0046] Mensah, G.A., Norrving, B., & Feigin, V.L. (2015). The global burden of stroke. *Neuroepidemiology*, 45(3), 143–145. 10.1159/000441082 26505979 PMC4631389

[CIT0047] Molamu, L. (1995). Wietie: The emergence and development of Tsotsitaal in South Africa. *Alternation*, 2(2), 139–158.

[CIT0048] Mosdell, J., Balchin, R., & Ameen, O. (2010). Adaptation of aphasia tests for neurocognitive screening in South Africa. *South African Journal of Psychology*, 40(3), 250–261. 10.1177/008124631004000304

[CIT0049] Myers-Scotton, C. (1997). *Duelling languages: Grammatical structure in codeswitching*. Clarendon Press.

[CIT0050] Myusken, P. (2000). *Bilingual speech: A typology of code-mixing*. Cambridge University Press.

[CIT0051] Odendaal, I., & Tönsing, K.M. (2024). Augmentative and alternative communication for individuals with post-stroke aphasia: Perspectives of South African speech-language pathologists. *Augmentative and Alternative Communication*, 1–9. 10.1080/07434618.2024.2374303 38995208

[CIT0052] Orlikoff, R.F., Schiavetti, N., & Metz, D.E. (2014). *Evaluating research in communicative disorders* (7th ed.). Pearson Communication Sciences and Disorders.

[CIT0053] Otheguy, R., García, O., & Reid, W. (2015). Clarifying translanguaging and deconstructing named languages: A perspective from linguistics. *Applied Linguistics Review*, 6(3), 281–307. 10.1515/applirev-2015-0014

[CIT0054] Paradis, M. (1987). *Bilingual aphasia test*. Lawrence Erlbaum Associates.

[CIT0055] Penn, C., & Beecham, R. (1992). Discourse therapy in multilingual aphasia: A case study. *Clinical Linguistics & Phonetics*, 6(1–2), 11–25. 10.3109/02699209208985516 20672881

[CIT0056] Pritchard, M., Hilari, K., Cocks, N., & Dipper, L. (2018). Psychometric properties of discourse measures in aphasia: Acceptability, reliability, and validity. *International Journal of Language and Communication Disorders*, 53(6), 1078–1093. 10.1111/1460-6984.12417 30155970

[CIT0057] Prust, M.L., Forman, R., & Ovbiagele, B. (2024). Addressing disparities in the global epidemiology of stroke. *Nature Reviews Neurology*, 20, 207–221. 10.1038/s41582-023-00921-z 38228908

[CIT0058] Ramos-Lima, M.J.M., Brasileiro, I.D.C., Lima, T.L.D., & Braga-Neto, P. (2018). Quality of life after stroke: Impact of clinical and sociodemographic factors. *Clinics*, 73, e418. 10.6061/clinics/2017/e418 30304300 PMC6152181

[CIT0059] Rodriguez-Fornells, A., Krämer, U.M., Lorenzo-Seva, U., Festman, J., & Münte, T.F. (2012). Self-assessment of individual differences in language switching. *Frontiers in Psychology*, 2, 388. 10.3389/fpsyg.2011.00388 22291668 PMC3254049

[CIT0060] Roushdy, T., Aref, H., Kesraoui, S., Temgoua, M., Nono, K.P., Gebrewold, M.A., Peter, W., Gopaul, U., Belahsen, M.F., Ben-Adji, D., Melifonwu, R., Pugazhendhi, S., Woodcock, N., Mohamed, M.H., Rossouw, A., Matuja, S., Ruanda, M.K., Mhiri, C., Saylor, D., Nahas, N.E. et al. (2022). Stroke services in Africa: What is there and what is needed. *International Journal of Stroke: Official Journal of the International Stroke Society*, 17(9), 972–982. 10.1177/17474930211066416 35034522

[CIT0061] Sefotho, M.P.M. (2022). Ubuntu translanguaging as a systematic approach to language teaching in multilingual classrooms in South Africa. *Journal for Language Teaching*, 56(1), 1–17. 10.56285/jltVol56iss1a5416

[CIT0062] Shiggins, C., Ryan, B., Dewan, F., Bernhardt, J., O’Halloran, R., Power, E., Lindley, R.I., McGurk, G., & Rose, M.L. (2024). Inclusion of people with aphasia in stroke trials: A systematic search and review. *Archives of Physical Medicine and Rehabilitation*, 105(3), 580–592. 10.1016/j.apmr.2023.06.010 37394026

[CIT0063] Simpson, J. (2017). Translanguaging in the contact zone: Language use in superdiverse urban areas. In H. Colman (Ed.), Multilingualisms and Development: Selected Proceedings of the 11th Language and Development Conference, New Delhi, India 2015. 11th Language and Development Conference: Multilingualism and Development (pp. 207–223). 18–20 November 2015, New Delhi: British Council India.

[CIT0064] Stark, B.C., Dutta, M., Murray, L.L., Fromm, D., Bryant, L., Harmon, T.G., Ramage, A.E., & Roberts, A.C. (2021). Spoken discourse assessment and analysis in aphasia: An international survey of current practices. *Journal of Speech, Language, and Hearing Research*, 64(11), 4366–4389. 10.1044/2021_JSLHR-20-00708 PMC9132151 34554878

[CIT0065] Treffers-Daller, J. (2024). Translanguaging: What is it besides smoke and mirrors? *Linguistic Approaches to Bilingualism*, 14(5), 1–26. 10.1075/lab.24015.tre

[CIT0066] Wagner, V.K., Ditsele, T., & Makgato, M.M. (2020). Influence of Sepitori on standard Setswana of its home language learners at three Tshwane townships. *Literator: Journal of Literary Criticism, Comparative Linguistics and Literary Studies*, 41(1), 1–7. 10.4102/lit.v41i1.1653

[CIT0067] Xin, S, Ping, W., & Qin, Y. (2021). Twenty years’ development of translanguaging: A bibliometric analysis. *International Journal of Multilingualism*, 21(1), 209–235. 10.1080/14790718.2021.2007933

